# Prevalence and factors associated with tobacco use among pregnant and breastfeeding mothers in India: insights from the National Family Health Survey-5 (2019–21)

**DOI:** 10.3389/fpubh.2025.1495522

**Published:** 2025-06-06

**Authors:** Rachita Pradhan, Rutuparna Sibani Dandsena, Sneha Singh, Shishirendu Ghosal, Debdutta Bhattacharya, Srikanta Kanungo, Sanghamitra Pati

**Affiliations:** ^1^ICMR-Regional Medical Research Centre, Bhubaneswar, India; ^2^Faculty of Biological Sciences, Academy of Scientific and Innovative Research (ACSIR), Bhubaneswar, India; ^3^Faculty of Medical Research, Academy of Scientific and Innovative Research (AcSIR), Bhubaneswar, India

**Keywords:** tobacco products, smokeless tobacco, pregnant women, breastfeeding, India, women of reproductive age, NFHS-5

## Abstract

**Background:**

Tobacco use remains a major public health concern, particularly in low- and middle-income countries, which account for 80% of the world’s tobacco users. In India, smokeless tobacco (SLT) use is higher among women, likely due to factors such as financial dependence resulting from limited occupational opportunities, peer influence, and gender inequality. Tobacco consumption in any form poses serious health risks to both mothers and their children.

**Objectives:**

This study aims to estimate the prevalence and factors associated with smoking tobacco and SLT among pregnant and breastfeeding mothers from the fifth National Family Health Survey (2019–21) (NFHS-5) in India.

**Methods:**

After excluding outliers, 722,933 women of reproductive age (24,368 pregnant, 102,080 breastfeeding, and 592,699 non-pregnant/non-breastfeeding mothers) were eligible for this cross-sectional secondary data analysis. Descriptive statistics are presented as means with standard deviations (continuous variables) or frequencies (categorical variables), with a 95% confidence interval as a measure of uncertainty. The magnitude of the influence on the covariates of tobacco consumption was estimated using univariate and multivariate logistic regressions.

**Results:**

Approximately 3.2% of mothers reported using tobacco during their current pregnancy or while breastfeeding, with a strong preference for SLT, which was over 13 times more common than smoking tobacco. Significant predictors of SLT consumption included tribal ethnicity [AOR: 2.20 (1.95–2.48)] and early motherhood [AOR: 1.12 (1.08–1.16)]. Notably, mass media awareness campaigns were ineffective in preventing SLT use among mothers [AOR: 1.49 (1.43–1.57)]. A marked difference in prevalence was observed between pregnant (2.48%) and breastfeeding (3.34%) mothers, suggesting a potential postpartum relapse. These findings underscore the potential role that Accredited Social Health Activists (ASHAs) can play during antenatal care (ANC) visits by providing consistent support for tobacco cessation.

**Conclusion:**

Tobacco use poses serious health hazards not only to the mother but also to the developing child. Our findings indicate a lack of management and awareness, underscoring the urgent need for reforms in tobacco production and distribution to ensure improved maternal care and child health outcomes.

## Introduction

Tobacco originated in the temperate regions of the United States (1, 2), where it was traditionally either smoked or chewed by Native Americans. In today’s world, smoking and smokeless tobacco are considered snuffs. Smoke from burning tobacco is drawn into the mouth and passed into the lungs, a method of consuming smoking tobacco (ST) (3). Smokeless tobacco (SLT), on the other hand, is used in several ways: dried tobacco leaves may be placed in the buccal sulcus, chewed, or sniffed through the nose, or less commonly (4), used as a dentifrice and for gargling (5).

The harmful effects of tobacco and its association with cardiovascular disease and cancer are no longer a mystery. Over the centuries, growing demand and easy availability have made tobacco a significant threat to mankind. This crisis is more confined to low- and middle-income countries (LMICs), where 80% of the global tobacco users reside (6). This issue is even more deeply rooted within eleven Southeast Asian countries, home to nearly 90% of the SLT users (5). Tobacco use, a major risk factor for chronic diseases and a leading cause of death in India, affects nearly 267 million adults and imposes a significant economic burden, costing the country over INR ₹177,000 crore (USD $27.5 billion) annually (ref-The WHO). Indian women consume more SLT products than men, probably due to financial dependence, lower educational attainment, lower engagement in different occupations, and gender inequality (7). Smoking tobacco products is costly and less socially acceptable. Additionally, many older women in India have a perception that SLT products are harmless (8). Our earlier finding reported that SLT consumption among women has reduced by more than 30%, but still, the prevalence is quite high (12.8%) (9).

Tobacco contains nicotine, which can pass through the placental barrier, leading to high nicotine concentrations in fetal serum and amniotic fluid, causing failed conception (10, 11). It is not at all favorable for fetal growth (it ends up in a fetus smaller for gestational age) (12), and can cause respiratory tract infection or asthma in newborns and children (13–16). Low birthweight (15, 17), preterm birth (18–20), stillbirth (19, 21, 22), otitis media (13), perinatal death, and sudden infant death syndrome (14, 15) are among other adverse effects of ST or SLT consumption during pregnancy.

The optimal period of exclusive breastfeeding is 6 months, which can be continued for another two years in parallel with family food (23). Women quit tobacco after cessation counseling during pregnancy, but relapse occurs during the postpartum period (24). As a result, nicotine and one of its by-products, cotinine, become available in breast milk (6). Among several unfavorable outcomes, reduced milk production due to prolactin shortage and shorter feeding time (25, 26); undesirable change in breastmilk composition (6, 13, 26–28) following a change in taste (26, 29), end up in delayed initiation of sucking reflex (13). Additionally, tobacco products being a neuro-stimulant, neonates of tobacco-consuming mothers remain more restless (23, 29), which might lead to poor learning ability and memory deficiency (30–32). All these factors altogether indicate poor breastfeeding habits, which ultimately lead to diminished immunity (6).

Tobacco is harmful not only to adults but also to a developing fetus in the womb and to a growing neonate who is dependent on breast milk. Although many mothers understand the risks of tobacco use and quit during pregnancy, significant inconsistency is evident among pregnant women in LMICs (6). Moreover, relapse has been reported in up to two-thirds of them within 6 months postpartum (33). A recent study based on breastfeeding mothers from 78 LMICs estimated the pooled prevalence of any form of tobacco (6.13%) and smokeless tobacco (4.92%) consumption to be highest from the South-East Asia Region (SEAR), and India is one among the top four countries in terms of the highest SLT use (6). Prevalence of SLT consumption among Indian pregnant women lies between 4% [National Family Health Survey-4 (2015–16)] and 7.4% [Global Adult Tobacco Survey-2 (GATS-2, 2016–17)], which was remarkably high among pregnant women aged between 25 and 29 years (34). To keep the mothers, as well as the infants, healthy, it is our duty to control unthoughtful tobacco consumption not only by the mothers but also in their surroundings. However, to date, no national-level information is available to indicate the same. Hence, this study aims to estimate the prevalence and its associated factors of tobacco consumption among childbearing and breastfeeding mothers from the recent National Family Health Survey-5 (NFHS-5, 2019–20) and compare the same with non-pregnant and non-breastfeeding women of reproductive age.

## Materials and methods

### Overview of data

The recent round of the National Family Health Survey, 2019–21 (NFHS-5), started in June 2019 and continued until April 2021. The key focus areas of this survey were maternal and child health, nutrition, and education. In the fifth edition, information was documented from 636,699 households, 724,115 women, 101,839 men, and 232,920 children through four survey schedules: household, women, men, and biomarkers. For this study, our focus was on the “woman’s schedule,” specifically, the health behavioral aspects of pregnant and breastfeeding mothers.

### Study design

The current study is based on the women’s dataset from the NFHS-5. As the data were collected cross-sectionally, the study design adopted was a cross-sectional analytical method.

### Inclusion and exclusion criteria

We estimated the highest and lowest ages of currently pregnant and breastfeeding mothers, respectively. The central 99th percentile of age (from the 0.5th percentile to the 99.5th percentile) of currently pregnant and breastfeeding mothers was included in the analysis. Observations belonging to the less than 0.5th percentile and more than 99.5th percentile were treated as outliers and excluded because the information may be less reliable from a cross-sectional secondary dataset. The remaining non-pregnant and non-breastfeeding mothers were included as comparators in the study population.

### Sample size

The NFHS-5 documented information on 724,115 women. After applying the inclusion criteria, 1,182 observations were excluded, resulting in a final sample size of 722,933.

### Independent variables

Age of women were categorized into four groups, namely “15–24 years,” “25–34 years,” “35–44 years,” and “more than 44 years.” Women’s educational levels were categorized into six groups based on their years of schooling. Those who never went to school were placed under “no formal education”; 1 to 5 completed years of schooling were placed under “up to primary education,” those receiving 6 to 8 years of institutional education were clubbed as “junior high,” followed by 9 to 10 years under “secondary education,” 11 to 12 under “higher secondary education,” and mothers attending school for more than 12 years were grouped into “Beyond Higher Secondary Education/Graduate and above.” Occupations of the mothers were organized into five categories: “currently not working,” “professional/sales/services,” “manual laborer,” and “agricultural laborer,” and the remaining less prevalent categories were clubbed together as “others.” States and union territories were categorized into six regions, viz. a viz., “north,” “east,” “west,” “south,” “central,” and “north-east” regions. Frequency of at least one in any of the following, among listening to radio, watching television, reading a newspaper or magazine less than once a week, or at least once a week, was considered to be some kind of exposure to media. The duration of tobacco use was expressed in weeks, months, and years and was converted to years only.

Mothers’ habit of smoking tobacco before their first pregnancy was estimated by deducting the age of their first child from the duration of tobacco product consumption. Mothers’ age at first delivery was calculated by deducting the ‘current age of first-born child’ from the ‘current age of that child’s mother’. Again, observations beyond the central 99th percentile were not considered in the analysis of this particular variable. As the current minimum legal age of marriage for women in India is 21 years, we further classified mothers’ age at first delivery into two categories: “lower age group,” where a woman became a mother before 21 years, and the other group consisted of those women who gave birth to a child at 21 years or at a higher age. Those women who responded affirmatively to alcohol consumption were further asked about their frequency of drinking. Based on these two questions, alcohol consumption was further grouped into three groups: “almost every day,” “does not consume regularly,” and “never consumes”.

### Outcome variable

The primary focus of this study was the consumption of tobacco products of any type. Additionally, we created two individual groups for smoking and smokeless tobacco. Information related to cigarettes, pipes, cigars, cheroots or cigarillos, water pipes/hookahs, and bidi smoking was grouped in the smoking tobacco group, while evidence in support of khaini, *paan* with tobacco, snuffs, and chewing tobacco consumption was collectively utilized to form the variable for smokeless tobacco.

### Statistical analysis

Data were analyzed using STATA version 16.0 (STATA Corp., Texas). A few of the observations contained missing or irrelevant information. They were ignored or treated as missing data wherever appropriate. To manage outliers, we retained observations within the central 99th percentile of the data and removed the top 0.5% and bottom 0.5% of extreme values. This approach helps minimize the influence of extreme outliers on the analysis while preserving the majority of the data distribution. The national-level sampling weight neutralized the differential probabilities of sample selection and ensured the generalizability of the study findings through the “*svyset*” command in STATA. Descriptive statistics in terms of mean with standard deviation and interquartile range (IQR) following the median represent the measures of central tendency and dispersion for a continuous variable, such as the age of women. For categorical variables, such as socio-demographic characteristics, the types of tobacco used were reported through frequency and proportion. Univariate binary logistic regression was used to assess the individual associations with smoking, smokeless, and any tobacco products. Variables found to be significantly associated in the univariate model were selected for the multivariate model. Associations from the regression models were expressed as odds ratios (ORs) with 95% confidence intervals (CIs).

### Sub-group analysis

Our target population, currently pregnant and breastfeeding mothers, was further analyzed as two mutually exclusive subsets. When the predictor variables were assessed only for pregnant mothers, women responding affirmatively to breastfeeding (in variable v404) were excluded. Similarly, for the breastfeeding subset of mothers who were currently pregnant (who responded yes in variable v213), mothers were omitted. Six separate multivariable logistic regression models were used to estimate the associated factors for three tobacco types in two of these subpopulations: currently pregnant and currently breastfeeding.

### Ethical consideration

During the NFHS-5, participants acknowledged their willingness to participate in the interviews and provided their consent before being interviewed. In this study, we used anonymized secondary data (NFHS-5), and there was no participant risk. The data used here are properly acknowledged and referenced, wherever required.

## Results

Among the mothers included from all over India, 18% were either pregnant or breastfeeding during the survey. The median age of the patients was 26 (IQR: 23–29) years. The majority of them were residents of a rural setup (80.7%), did not engage in any livelihood activities (83%), and were reported to be exposed to mass media, providing information regarding the harmful effects of tobacco (71.5%). A considerable proportion of mothers (around 47%) reported having their first child between the ages of 13 and 20, which is below the current legal age of marriage in India (21 years). A detailed description of the study sample is provided in the Supplementary Table S1.

Overall, tobacco use was reported by 3.2% of mothers, with a lower prevalence among currently pregnant (2.48%) and breastfeeding mothers (3.34%) compared to those who were neither pregnant nor breastfeeding (4.37%). Bidi (0.06% among pregnant and 0.09% among lactating women)—a small cigarette without a filter, filled with unprocessed dried tobacco flakes, wrapped in a tendu leaf tied with a string or adhesive—and *paan* with tobacco (0.61% in pregnant and 0.81% in lactating), and betel leaf preparation containing areca nut and lime were the two most chosen tobacco products from the smoking and SLT categories, respectively (Table 1).

**Table 1:** Prevalence of various tobacco product use among the two groups (currently pregnant and/or breastfeeding mothers and women who are not pregnant at the time of interview).

Types of tobacco products		Currently pregnant mothers *n*; % (95% CI)	Currently breastfeeding mothers *n*; % (95% CI)	Currently non-pregnant and non-breastfeeding mothers *n*; % (95% CI)
Use of any tobacco	Yes	573; 2.48 (2.28–2.70)	3,334; 3.34 (3.23–3.46)	26,055; 4.37 (4.31–4.41)
	No	22,516; 97.52 (97.31–97.71)	96,361; 96.66 (96.54–96.77)	570,307; 95.63 (95.59–95.69)
Form of tobacco	Smoking only (*n* = 2,728)	37; 0.16 (0.11–0.22)	231; 0.23 (0.20–0.26)	2,460; 0.41 (0.39–0.43)
	Smokeless only (*n* = 25,347)	492; 2.13 (1.95–2.33)	2,898; 2.91 (2.80–3.01)	21,956; 3.67 (3.62–3.71)
Smoking tobacco	Cigarette (*n* = 745)	11; 0.05 (0.02–0.09)	66; 0.07 (0.05–0.09)	6; 0.11 (0.10–0.12)
	Bidi (*n* = 1,303)	14; 0.06 (0.03–0.10)	88; 0.09 (0.07–0.11)	1,201; 0.20 (0.18–0.21)
	Cigar (*n* = 323)	8; 0.03 (0.01–0.06)	22; 0.02 (0.01–0.03)	293; 0.05 (0.04–0.06)
	Pipe (*n* = 222)	5; 0.02 (0.01–0.05)	26; 0.03 (0.02–0.04)	191; 0.03 (0.02–0.04)
	Hookah (*n* = 519)	6; 0.03 (0.01–0.06)	56; 0.06 (0.04–0.07)	457; 0.08 (0.07–0.09)
Smokeless tobacco	Khaini (*n* = 6,378)	122; 0.53 (0.43–0.63)	742; 0.74 (0.69–0.80)	5,514; 0.92 (0.90–0.95)
	*Paan* with tobacco (*n* = 8,136)	140; 0.61 (0.51–0.71)	810; 0.81 (0.76–0.87)	7,186; 1.20 (1.17–1.22)
	Other chewing tobacco (*n* = 2,372)	52; 0.22 (0.17–0.30)	244; 0.24 (0.22–0.28)	2076; 0.35 (0.33–0.36)
	Snuff (*n* = 751)	3; 0.01 (0.01–0.03)	67; 0.07 (0.05–0.09)	681; 0.11 (0.10–0.12)

**Figure 1:**
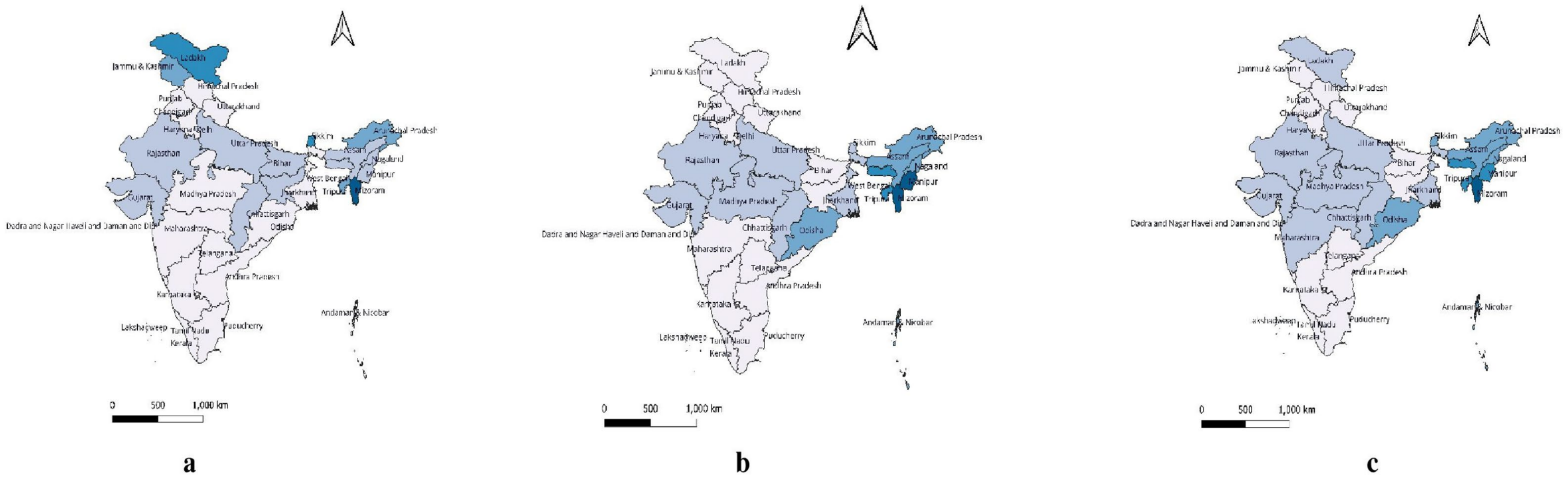
State-wise prevalence of **(a)** smoking tobacco, **(b)** smokeless tobacco and **(c)** any-tobacco consumption among the mothers who were pregnant or lactating at the time of interview.

Smoking and smokeless tobacco products consumed by pregnant and breastfeeding mothers were distributed distinctively among different socio-economic groups (Table 2). The use of SLT was much higher (8.05%) among tribal women than among other ethnic groups (scheduled caste: 2.62%; OBC: 1.85%; others: 1.63%). Similarly, the use of both smoking and SLT products was higher among working mothers (5.40%) than among non-working mothers (2.63%). Mothers from the weaker economic classes consumed more tobacco products than those from the middle and affluent classes. For weaker economic classes, we refer to households belonging to the lower wealth quintiles (Q1 and Q2), which represent the bottom 40% of the population in terms of asset-based wealth, as categorized by the NFHS. Smoking and smokeless were both quite common among the northeastern states, followed by the central Indian states. Both smoking and SLT were fairly common with the habit of drinking alcohol. Pregnant or breastfeeding mothers with exposure to newspapers, television, or radio were found to be unlikely to smoke tobacco (0.15%), while the share of SLT users was relatively higher (2.21%). The detailed state/union territory-wise prevalence of tobacco consumption is shown in Figure 1.

**Table 2:** Prevalence of various tobacco products among different socio-demographic groups.

Socio-demographic and behavioral covariates		Currently Pregnant and/or Breastfeeding mothers [*n*, % (95% CI)]		
		Smoking tobacco	Smokeless tobacco	Any tobacco product
Mothers age at first delivery	Lower age group (13–20 years) (*n* = 61,049)	152; 0.25 (0.21–0.29)	1913; 3.13 (2.99–3.27)	2,201; 3.61 (3.45–3.75)
	Higher age group (21–45 years) (*n* = 69,080)	128; 0.18 (0.15–0.22)	1,684; 2.44 (2.32–2.55)	1941; 2.81 (2.68–2.93)
Residence	Rural (*n* = 97,451)	243; 0.25 (0.21–0.28)	3,067; 3.15 (3.03–3.25)	3,550; 3.64 (3.52–3.76)
	Urban (*n* = 32,783)	37; 0.11 (0.07–0.15)	539; 1.64 (1.50–1.78)	601; 1.83 (1.69–1.98)
Education	No formal education (*n* = 25,008)	153; 0.61 (0.51–0.71)	1,570; 6.28 (5.97–6.58)	1798; 7.19 (6.86–7.51)
	Primary (*n* = 14,986)	42; 0.28 (0.20–0.37)	731; 4.88 (4.53–5.23)	844; 5.63 (5.26–6.00)
	Junior High (*n* = 22,593)	30; 0.13 (0.08–0.18)	663; 2.93 (2.71–3.16)	764; 3.38 (3.14–3.62)
	Secondary (*n* = 26,831)	23; 0.08 (0.05–0.12)	458; 1.71 (1.55–1.86)	526; 1.96 (1.79–2.13)
	Higher Secondary (*n* = 18,991)	20; 0.11 (0.06–0.16)	132; 0.69 (0.58–0.82)	155; 0.82 (0.69–0.95)
	Above higher secondary (*n* = 21,825)	11; 0.05 (0.02–0.09)	51; 0.23 (0.17–0.30)	65; 0.30 (0.22–0.37)
Ethnicity	Scheduled caste (*n* = 30,740)	82; 0.27 (0.21–0.33)	806; 2.62 (2.44–2.80)	938; 3.05 (2.86–3.24)
	Scheduled tribe (*n* = 13,697)	55; 0.40 (0.30–0.52)	1,102; 8.05 (7.59–8.51)	1,283; 9.36 (8.88–9.86)
	Other backward classes (*n* = 55,474)	104; 0.19 (0.15–0.22)	1,024; 1.85 (1.73–1.96)	1,179; 2.13 (2.00–2.24)
	Others (*n* = 22,660)	28; 0.12 (0.08–0.17)	370; 1.63 (1.47–1.80)	420; 1.85 (1.68–2.03)
Occupation	Currently working (*n* = 2,810)	14; 0.49 (0.24–0.78)	151; 5.40 (4.56–6.27)	182; 6.50 (5.59–7.45)
	Currently not working (*n* = 16,783)	28; 0.16 (0.11–0.24)	441; 2.63 (2.39–2.88)	500; 2.98 (2.72–3.24)
Wealth Index	Poorest (*n* = 32,502)	159; 0.49 (0.41–0.57)	1975; 6.08 (5.81–6.34)	2,272; 6.99 (6.71–7.27)
	Poorer (*n* = 28,969)	41; 0.14 (0.10–0.19)	884; 3.05 (2.85–3.25)	992; 3.43 (3.21–3.64)
	Middle (*n* = 25,409)	45; 0.18 (0.12–0.23)	469; 1.84 (1.68–2.01)	557; 2.19 (2.01–2.37)
	Richer (*n* = 23,665)	17; 0.07 (0.04–0.11)	203; 0.86 (0.74–0.98)	238; 1.01 (0.08–1.14)
	Richest (*n* = 19,689)	17; 0.09 (0.05–0.13)	72; 0.37 (0.28–0.46)	91; 0.46 (0.37–0.56)
Region	North (*n* = 8,728)	21; 0.24 (0.14–0.36)	21; 0.25(0.14–0.36)	43; 0.49 (0.35–0.66)
	Central (*n* = 44,434)	124; 0.28 (0.23–0.33)	1,512; 3.40 (3.23–3.57)	1713; 3.86 (3.67–4.04)
	East (*n* = 37,798)	64; 0.17 (0.13–0.21)	737; 1.95 (1.81–2.09)	883; 2.34 (2.18–2.49)
	North-east (*n* = 5,881)	32; 0.55 (0.37–0.76)	819; 13.94 (13.06–14.85)	873; 14.84 (13.94–15.77)
	West (*n* = 14,573)	26; 0.18 (0.11–0.26)	451; 3.10 (2.81–3.38)	555; 3.81 (3.50–4.13)
	South (*n* = 18,820)	12; 0.07 (0.03–0.11)	62; 0.33 (0.25–0.42)	83; 0.44(0.35–0.54)
Alcohol consumption	Almost every day (*n* = 116)	3; 2.82 (0.53–7.37)	43; 37.83 (29.08–47.40)	45; 39.15 (29.88–48.28)
	Not consumes regularly (*n* = 592)	22; 3.88 (2.34–5.57)	188; 31.74 (28.02–35.67)	222; 37.54 (33.58–41.53)
	Never consumes (*n* = 129,526)	253; 0.20(0.17–0.22)	3,373; 2.60 (2.51–2.69)	3,883; 3.00 (2.90–3.09)
Exposure to mass-media	To some extent (*n* = 93,517)	141; 0.15 (0.12–0.17)	2065; 2.21 (2.11–2.30)	2,365; 2.53 (2.42–2.63)
	Not at all (*n* = 36,717)	139; 0.38 (0.31–0.44)	1,540; 4.19 (3.99–4.40)	1786;4.86(4.64–5.08)

The likelihood of smoking tobacco products among Indian women increased with age, early motherhood, none-to-minimal educational attainment, poverty, and alcohol consumption (Table 3). Similar findings were also evident among SLT users. The difference was found for the covariate of mass media, which emerged as a protective factor against smoking tobacco, though insignificantly [AOR: 0.90 (0.79–1.01)]; odds of SLT consumption were significantly higher among mothers exposed to media components [AOR: 1.49 (1.43–1.57)].

**Table 3:** Multivariable association of various tobacco products with socio-demographic and behavioral covariates among women of reproductive age.

Socio-demographic and behavioral covariates		Odds ratio (95% CI)		
		Smoking tobacco	Smokeless tobacco	Any-tobacco
Age category	15–24 years	Reference		
	25–34 years	1.24 (1.00–1.53)	2.39 (2.23–2.56)	2.28 (2.13–2.43)
	35–44 years	2.02 (1.56–2.60)	3.50 (3.24–3.78)	3.47 (3.21–3.73)
	>44 years	3.15 (2.40–4.12)	4.29 (3.94–4.66)	4.39 (4.03–4.76)
Age at first delivery	Lower age group (13–20 years)	1.13 (1.02–1.27)	1.11 (1.06–1.15)	1.12 (1.08–1.16)
	Higher age group (21–45 years)	Reference		
Residence	Urban	0.96 (0.81–1.14)	1.29 (1.19–1.39)	1.24 (1.16–1.34)
	Rural	Reference		
Education	No formal education	2.69 (1.97–3.64)	10.00 (8.66–11.54)	7.95 (6.68–9.47)
	Primary	1.30 (0.092–1.84)	9.16 (7.96–10.52)	7.11 (5.98–8.44)
	Junior High	0.69 (0.48–0.99)	5.71 (4.96–6.57)	4.35 (3.64–5.19)
	Secondary	0.60 (0.40–0.90)	3.25 (2.82–3.73)	2.59 (2.16–3.11)
	Higher Secondary	0.81 (0.55–1.20)	1.77 (1.51–2.06)	1.48 (1.23–1.77)
	Above higher secondary	Reference		
Ethnicity	Scheduled Tribe	1.09 (0.92–1.30)	2.06 (1.94–2.20)	2.07 (1.96–2.19)
	Scheduled Caste	1.30 (1.13–1.49)	1.41 (1.32–1.48)	1.40 (1.32–1.47)
	Other Backward Class	Reference		
	None of them	1.04 (0.83–1.31)	1.21 (1.13–1.30)	1.20 (1.12–1.27)
Wealth index	Poorest	1.71 (1.20–2.43)	4.56 (4.00–5.20)	4.08 (3.59–4.64)
	Poorer	1.46 (1.07–1.99)	3.21 (2.83–3.65)	2.91 (2.57–3.29)
	Middle	1.35 (1.00–1.83)	2.43 (2.15–2.75)	2.23 (1.97–2.51)
	Richer	0.84 (0.60–1.15)	1.64 (1.44–1.86)	1.45 (1.28–1.65)
	Richest	Reference		
Region	Central	0.66 (0.52–0.82)	7.53 (6.17–9.20)	3.50 (3.11–3.94)
	East	0.40 (0.28–0.58)	6.14 (5.01–7.53)	2.84 (2.49–3.23)
	North-east	1.30 (0.99–1.70)	36.43 (29.67–44.72)	15.27 (13.44–17.34)
	West	0.30 (0.21–0.43)	9.54 (7.75–11.76)	4.70 (4.11–5.37)
	South	0.29 (0.22–0.37)	2.92 (2.37–3.60)	1.33 (1.16–1.52)
	North	Reference		
Frequency of alcohol consumption	Almost every day	5.21 (3.09–8.79)	7.09 (5.85–8.59)	6.91 (5.66–8.44)
	Not consumes regularly	7.74 (5.88–10.19)	4.46 (3.99–4.99)	5.55 (4.96–6.22)
	Never consumes	Reference		
Exposure to mass media	To some extent	0.90 (0.79–1.01)	1.49 (1.43–1.57)	1.46 (1.40–1.53)
	Not at all	Reference		
Currently pregnant and breastfeeding mother	Yes	0.79 (0.65–0.96)	0.95 (0.90–1.00)	0.94 (0.90–0.99)
	No	Reference		

We further estimated the association of possible predictors of tobacco consumption during pregnancy and breastfeeding separately. Major differences were found only in the age and educational attainment of mothers (Table 4). The odds of smokeless tobacco consumption among pregnant mothers were relatively low in the subset of breastfeeding mothers across all age groups. Higher odds of any tobacco consumption were reported at higher ages [AOR at 35–44 years: 4.05 (3.70–4.43) and the highest age group of >44 years: 5.08 (4.61–5.59)] compared to the lower age strata (15–24 years). An opposite association was observed with the educational attainment of the mother, where least educated pregnant mothers showed higher odds of SLT consumption than breastfeeding mothers with equivalent academic qualifications [AOR 9.38 (8.04–10.94) vs. 9.10 (7.79–10.62) for the mother with no formal education], and a downward trend for AOR was observed with academic growth.

**Table 4:** Association of various covariates with various tobacco products among currently pregnant mothers and comparison with currently breastfeeding mothers.

Socio-demographic characteristics		Odds ratio (95% CI)					
		Only pregnant mothers			Only breastfeeding mothers		
		Smoking tobacco	Smokeless tobacco	Any-tobacco	Smoking tobacco	Smokeless tobacco	Any-tobacco
Age category	15–24 years	Reference					
	25–34 years	1.21 (0.95–1.54)	2.82 (2.59–3.07)	2.63 (2.43–2.85)	1.22 (0.95–1.58)	2.93 (2.68–3.20)	2.73 (2.51–2.97)
	35–44 years	2.01 (1.53–2.62)	4.05 (3.69–4.43)	3.93 (3.60–4.28)	1.96 (1.48–2.60)	4.19 (3.81–4.60)	4.05 (3.70–4.43)
	>44 years	3.15 (2.38–4.17)	4.91 (4.46–5.41)	4.93 (4.49–5.41)	3.09 (2.31–4.15)	5.08 (4.60–5.61)	5.08 (4.61–5.59)
Age at first delivery	Lower age group (13–20 years)	1.14 (1.02–1.28)	1.12 (1.07–1.16)	1.13 (1.09–1.17)	1.13 (1.01–1.27)	1.11 (1.07–1.16)	1.13 (1.08–1.17)
	Higher age group (21–45 years)	Reference					
Residence	Urban	0.98 (0.82–1.17)	1.28 (1.18–1.38)	1.24 (1.15–1.33)	0.97 (0.81–1.16)	1.27 (1.17–1.37)	1.23 (1.14–1.33)
	Rural	Reference					
Education	No formal education	2.55 (1.86–3.49)	9.38 (8.04–10.94)	7.42 (6.14–8.95)	2.51 (1.83–3.44)	9.10 (7.79–10.62)	7.17 (5.92–8.68)
	Primary	1.22 (0.85–1.74)	8.73 (7.53–10.14)	6.69 (5.55–8.06)	1.20 (0.83–1.72)	8.54 (7.34–9.93)	6.51 (5.39–7.87)
	Junior High	0.64 (0.44–0.93)	5.50 (4.74–6.38)	4.11 (3.39–4.97)	0.64 (0.44–0.93)	5.39 (4.63–6.26)	4.01 (3.30–4.88)
	Secondary	0.58 (0.38–0.88)	3.09 (2.66–3.58)	2.44 (2.00–2.97)	0.57 (0.38–0.88)	3.04 (2.62–3.54)	2.40 (1.96–2.93)
	Higher Secondary	0.76 (0.51–1.14)	1.72 (1.46–2.03)	1.43 (1.17–1.74)	0.75 (0.50–1.13)	1.70 (1.43–2.01)	1.41 (1.15–1.73)
	Above higher secondary	Reference					
Ethnicity	Scheduled Tribe	1.09 (0.91–1.30)	2.03 (1.90–2.17)	2.03 (1.91–2.15)	1.11 (0.93–1.32)	2.04 (1.90–2.18)	2.03 (1.91–2.16)
	Scheduled Caste	1.30 (1.12–1.50)	1.41 (1.33–1.50)	1.40 (1.33–1.48)	1.31 (1.13–1.51)	1.42 (1.34–1.51)	1.41 (1.33–1.49)
	Other Backward Class	Reference					
	None of them	1.05 (0.83–1.34)	1.21 (1.13–1.30)	1.20 (1.12–1.29)	1.06 (0.84–1.35)	1.22 (1.13–1.31)	1.20 (1.12–1.30)
Wealth index	Poorest	1.66 (1.14–2.40)	4.50 (3.94–5.15)	4.02 (3.52–4.58)	1.61 (1.10–2.34)	4.48 (3.93–5.11)	4.00 (3.50–4.55)
	Poorer	1.52 (1.10–2.10)	3.20 (2.81–3.64)	2.91 (2.56–3.30)	1.51 (1.09–2.10)	3.22 (2.83–3.66)	2.92 (2.58–3.32)
	Middle	1.37 (1.00–1.87)	2.42 (2.13–2.75)	2.21 (1.95–2.50)	1.34 (0.98–1.84)	2.42 (2.14–2.74)	2.21 (1.95–2.50)
	Richer	0.87 (0.62–1.21)	1.64 (1.44–1.86)	1.45 (1.28–1.65)	0.86 (0.61–1.20)	1.65 (1.45–1.87)	1.46 (1.28–1.66)
	Richest	Reference					
Region	Central	0.66 (0.52–0.83)	7.36 (6.02–9.00)	3.40 (3.02–3.83)	0.67 (0.53–0.84)	7.38 (6.05–9.01)	3.40 (3.02–3.84)
	East	0.41 (0.28–0.61)	6.46 (5.26–7.94)	2.94 (2.58–3.35)	0.43 (0.29–0.63)	6.60 (5.38–8.11)	3.00 (2.63–3.42)
	North-east	1.30 (0.98–1.72)	36.64 (29.78–45.08)	15.11 (13.26–17.21)	1.29 (0.98–1.71)	36.57 (29.74–44.95)	15.02 (13.17–17.13)
	West	0.28 (0.20–0.39)	9.34 (7.57–11.53)	4.53 (3.96–5.19)	0.28 (0.20–0.40)	9.31 (7.55–11.48)	4.52 (3.94–5.18)
	South	0.28 (0.22–0.37)	2.96 (2.40–3.65)	1.31 (1.16–1.51)	0.29 (0.22–0.38)	2.99 (2.43–3.69)	1.33 (1.17–1.53)
	North	Reference					
Frequency of alcohol consumption	Almost every day	5.27 (3.04–9.15)	6.84 (5.59–8.36)	6.75 (5.47–8.33)	5.19 (2.99–9.02)	6.87 (5.61–8.41)	6.80 (5.50–8.40)
	Not consumes regularly	7.86 (5.90–10.49)	4.42 (3.93–4.97)	5.54 (4.91–6.24)	7.84 (5.87–10.48)	4.39 (3.91–4.94)	5.51 (4.89–6.21)
	Never consumes	Reference					
Exposure to mass media	To some extent	0.90 (0.80–1.02)	1.50 (1.43–1.58)	1.46 (1.40–1.53)	0.89 (0.79–1.01)	1.50 (1.43–1.58)	1.46 (1.40–1.53)
	Not at all	Reference					

**Figure 2:**
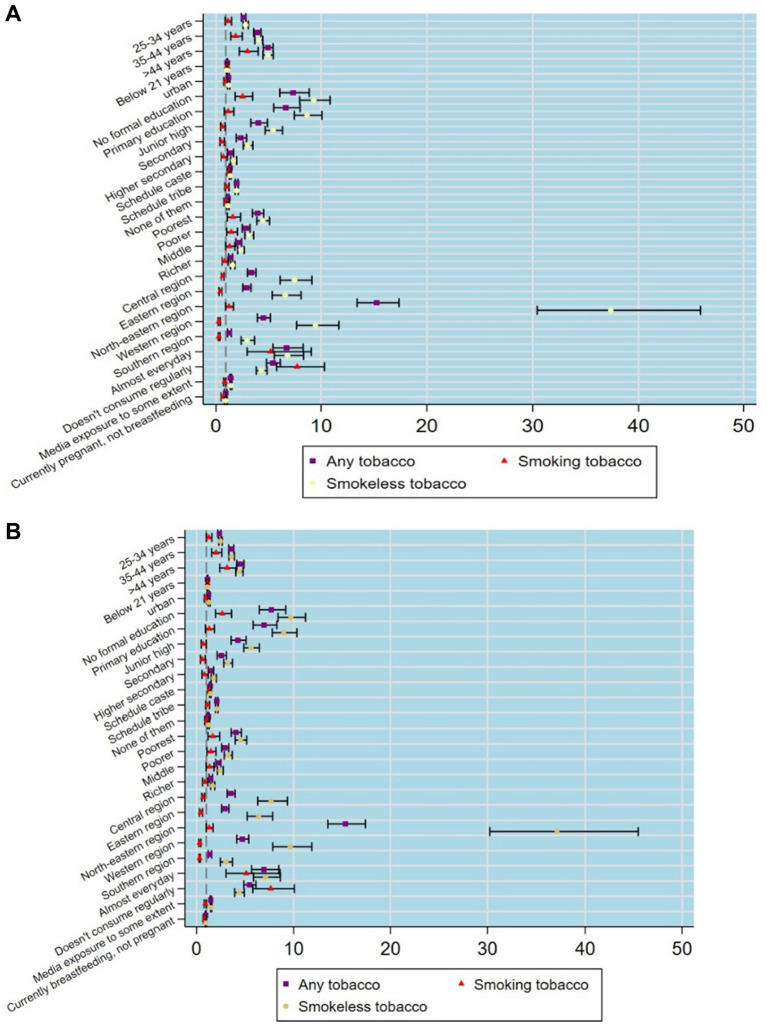
**(A)** Tobacco use by socio-demographic characteristics among currently pregnant women based on logistic regression estimates. **(B)** Tobacco use by socio-demographic characteristics among currently breastfeeding women based on logistic regression estimates.

Logistic regression (Figures 2A,B) indicates that tobacco use, especially smokeless forms, is higher among both currently pregnant and breastfeeding women who are less educated, belong to Scheduled Tribes, are in the poorest wealth quintile, and reside in the north-eastern regions.

When comparing the two groups (currently pregnant or breastfeeding and currently non-pregnant or not breastfeeding), the odds of smoking tobacco consumption among pregnant and breastfeeding mothers were found to be higher than those of non-pregnant or non-breastfeeding mothers with increasing age (Table 5). The likelihood of SLT use among pregnant/breastfeeding mothers from urban [AOR: 1.49 (1.25–1.76)] setups was greater in comparison to the non-pregnant or non-breastfeeding rural women. Odds for smoking and SLT consumption were higher among all subcategories of educational attainment and six national regions for the currently pregnant/breastfeeding women. Odds of SLT consumption among the pregnant/breastfeeding tribal women were found to be higher [AOR: 2.20 (1.95–2.48)] among scheduled tribe women than the non-pregnant/non-breastfeeding women [AOR: 2.03 (1.90–2.18)] with respect to the women from other backward classes. Early motherhood appeared as a risk factor [AOR: 1.38 (1.00–1.91)] for tobacco smoking among pregnant/breastfeeding mothers. In addition, exposure to mass media was also found to be associated with higher odds of smokeless tobacco consumption [AOR: 1.44 (1.30–1.60)] during pregnancy or breastfeeding. The habit of alcohol consumption during pregnancy or nursing was also found to be significantly associated with tobacco consumption.

**Table 5:** Comparison of the association between various tobacco products and socio-demographic covariates between currently pregnant/breastfeeding mothers and non-pregnant and non-breastfeeding women.

Socio-demographic and behavioral covariates		Odds Ratio (95% CI)					
		Smoking tobacco		Smokeless tobacco		Any-tobacco	
		Currently pregnant or breastfeeding	Non-pregnant/non-breastfeeding	Currently pregnant or breastfeeding	Non-pregnant/non-breastfeeding	Currently pregnant or breastfeeding	Non-pregnant/non-breastfeeding
Age category	15–24 years	Reference					
	25–34 years	1.43 (1.01–2.04)	1.23 (0.95–1.59)	1.44 (1.30–1.60)	2.98 (2.73–3.26)	1.45 (1.31–1.60)	2.78 (2.55–3.02)
	35–44 years	3.29 (1.98–5.47)	1.94 (1.46–2.57)	1.79 (1.54–2.09)	4.25 (3.87–4.68)	1.89 (1.64–2.20)	4.11 (3.75–4.49)
	>44 years	—	3.05 (2.27–4.10)	—	5.16 (4.67–5.70)	—	5.15 (4.68–5.67)
Age at first delivery	Lower age group (13–20 years)	1.38 (1.00–1.91)	1.12 (1.00–1.26)	1.05 (0.96–1.14)	1.11 (1.07–1.16)	1.06 (0.97–1.15)	1.13 (1.08–1.17)
	Higher age group (21–45 years)	Reference					
Residence	Urban	0.83 (0.46–1.49)	0.97 (0.81–1.16)	1.49 (1.25–1.76)	1.27 (1.17–1.37)	1.37 (1.16–1.60)	1.23 (1.14–1.33)
	Rural	Reference					
Education	No formal education	4.72 (2.15–10.33)	2.53 (1.85–3.47)	13.58 (9.78–18.85)	9.02 (7.72–10.54)	11.97 (8.94–16.02)	7.11 (5.87–8.60)
	Primary	2.71 (1.21–6.11)	1.21 (0.84–1.73)	10.07 (7.27–13.96)	8.48 (7.29–9.87)	9.13 (6.83–12.20)	6.47 (5.36–7.82)
	Junior High	1.46 (0.66–3.22)	0.64 (0.44–0.93)	6.42 (4.62–8.93)	5.36 (4.61–6.23)	5.79 (4.33–7.75)	3.99 (3.28–4.84)
	Secondary	1.05 (0.47–2.35)	0.57 (0.37–0.87)	4.16 (3.00–5.78)	3.03 (2.61–3.53)	3.74 (2.79–5.02)	2.39 (1.95–2.92)
	Higher Secondary	1.70 (0.75–3.90)	0.75 (0.50–1.14)	2.18 (1.55–3.07)	1.69 (1.43–2.01)	1.99 (1.46–2.70)	1.41 (1.15–1.72)
	Above higher secondary	Reference					
Ethnicity	Scheduled Tribe	0.93 (0.60–1.42)	1.11 (0.93–1.33)	2.20 (1.95–2.48)	2.03 (1.90–2.18)	2.26 (2.02–2.53)	2.03 (1.91–2.16)
	Scheduled Caste	1.23 (0.85–1.78)	1.31 (1.13–1.52)	1.28 (1.13–1.45)	1.42 (1.34–1.51)	1.29 (1.14–1.45)	1.41 (1.34–1.49)
	Other Backward Class	Reference					
	None of them	0.83 (0.46–1.48)	1.07 (0.84–1.36)	1.17 (0.99–1.39)	1.22 (1.13–1.31)	1.14 (0.97–1.35)	1.20 (1.12–1.29)
Wealth index	Poorest	2.50 (1.01–6.16)	1.61 (1.10–2.34)	5.71 (3.73–8.76)	4.47 (3.92–5.10)	5.40 (3.72–7.84)	3.99 (3.50–4.55)
	Poorer	1.02 (0.42–2.45)	1.51 (1.09–2.09)	3.49 (2.30–5.29)	3.21 (2.83–3.65)	3.20 (2.23–4.61)	2.92 (2.58–3.31)
	Middle	1.60 (0.65–3.94)	1.34 (0.97–1.83)	2.70 (1.83–3.98)	2.41 (2.13–2.73)	2.64 (1.87–3.71)	2.20 (1.95–2.49)
	Richer	0.67 (0.28–1.64)	0.86 (0.61–1.20)	1.61 (1.07–2.43)	1.65 (1.45–1.87)	1.54 (1.07–2.21)	1.45 (1.28–1.65)
	Richest	Reference					
Region	North	3.13 (1.47–6.65)	3.43 (2.63–4.48)	0.74 (0.43–1.28)	0.33 (0.27–0.403)	1.11 (0.72–1.72)	0.74 (0.65–0.85)
	Central	1.92 (0.97–3.82)	2.29 (1.85–2.85)	5.47 (4.17–7.18)	2.46 (2.25–2.69)	4.61 (3.53–6.00)	2.55 (2.34–2.77)
	East	0.93 (0.44–1.96)	1.46 (1.07–2.01)	2.32 (1.74–3.11)	2.22 (2.01–2.45)	2.07 (1.56–2.74)	2.26 (2.05–2.49)
	North-east	4.19 (2.04–8.62)	4.46 (3.41–5.84)	21.99 (16.64–29.07)	12.23 (11.05–13.54)	17.51 (13.33–23.00)	11.27 (10.21–12.44)
	West	2.25 (0.88–5.75)	0.96 (0.67–1.38)	7.25 (5.31–9.91)	3.10 (2.77–3.46)	6.71 (4.94–9.11)	3.37 (3.03–3.75)
	South	Reference					
Frequency of alcohol consumption	Almost every day	6.68 (2.24–19.93)	5.16 (2.97–8.960)	10.34 (6.67–16.01)	6.86 (5.60–8.40)	8.99 (5.83–13.86)	6.78 (5.49–8.39)
	Not consumes regularly	8.36 (4.25–16.45)	7.74 (5.79–10.33)	5.13 (4.05–6.49)	4.33 (3.86–4.87)	6.13 (4.82–7.78)	5.43 (4.81–6.12)
	Never consumes	Reference					
Exposure to mass media	To some extent	1.20 (0.70–1.48)	0.89 (0.79–1.007)	1.44 (1.30–1.602)	1.50 (1.43–1.58)	1.42 (1.29–1.57)	1.46 (1.40–1.53)
	Not at all	Reference					

## Discussion

This study provided a holistic view of the magnitude of tobacco consumption among pregnant and breastfeeding mothers and identified the degree of influence of various covariates at the national level. A few important insights we observed were that familiarity with smokeless tobacco among women was greater than that with smoking tobacco products in India. Even in some of the socio-demographic sub-groups, the odds of tobacco consumption were relatively higher among pregnant and/or breastfeeding mothers than among non-pregnant/non-breastfeeding women. The tribal population and mothers belonging to the regressive classes from the asset index showed a high likelihood of tobacco product use. Both types of tobacco use were prominently associated with mothers from the northeastern states. Early pregnancy appeared as a risk factor for mothers to use tobacco, while mass media acted as a protective factor for smoking tobacco only, providing no defense against the use of smokeless tobacco products during pregnancy and breastfeeding.

SLT products are relatively common among women from India for a few reasons, such as being more socially acceptable for women (35), relatively affordable (36) for women when they are more financially dependent on their spouses, easily available in the remotest areas (35), and in several states, women are the sole workers in the tobacco industry (37), which makes tobacco easily accessible to them. In many communities, SLT is regarded as a traditional remedy for digestive issues, nausea, and dental health. It is believed that certain forms of tobacco help alleviate pregnancy-related discomforts such as morning sickness and acidity. Due to these deeply embedded beliefs, SLT use is normalized, making it challenging to deter its consumption among pregnant mothers (38). In households where tobacco use is customary, young women may adopt the habit early on and continue its use during pregnancy, unaware or unconcerned about risks. When a newlywed comes to a tobacco-infiltrated family, her affinity for tobacco might occur from peer pressure or influence from other family members (39, 40) as SLT is considered less harmful than smoking tobacco (41). Unlike smoking, which carries a visible social and cultural taboo, SLT use is discreet and allows women to consume it with minimal scrutiny. As a result, SLT serves as a socially sanctioned alternative for women of all age groups (42). Studies have reported that women perceive SLT as less harmful than smoking, and cultural beliefs around its medicinal properties during pregnancy further normalize its use (43, 44).

Pregnant and breastfeeding women with no formal education and relatively fewer years of formal education, a habit of alcohol consumption, and from less affluent households showed a higher likelihood of smoking tobacco as well as SLT (1,2) (45, 46). These findings depict that dependency on alcohol before getting pregnant might not be easy to overcome during pregnancy or breastfeeding. Girls who marry and conceive early are often deprived of formal education and are exposed to high levels of stress and social pressure, which can make them more vulnerable to adopting maladaptive coping strategies such as tobacco use. Low educational attainment contributes to poor health literacy and reduced awareness of the risks associated with tobacco use during pregnancy and breastfeeding. It may also reflect broader socio-economic disadvantages, which restrict access to healthcare and increase vulnerability to social norms that normalize smokeless tobacco use among women. These women may also have lower decision-making autonomy and reduced exposure to mass media or institutional health messaging, limiting opportunities for behavioral change. In the Indian health system, once the pregnancy is confirmed, it is registered by an ASHA worker who generally counsels the expecting parents on several pregnancy-related risk behaviors and how to overcome them; getting rid of tobacco and alcohol habits is two of them (47). It was also found that the odds of SLT use were consistently higher among pregnant and breastfeeding mothers than among non-pregnant and non-breastfeeding mothers at all levels of educational attainment. Even highly educated mothers were unable to internalize the counseling provided during pregnancy and childbirth. Therefore, the questions that arise here are, “Why are they ignoring the advice from the health workers?,” or “Are they too addicted to stop tobacco?,” “Why does not higher education act as a barrier to tobacco consumption?,” “Is higher education failing to empower women to stop consuming tobacco?.” To find an appropriate answer, a survey such as the Global Adult Tobacco Survey needs to be repeated at regular intervals. Additionally, findings that cannot be assessed through a quantitative survey or a qualitative or mixed-method approach can be adopted.

The geographical territories of the Indian subcontinent are also characterized by their socio-cultural differences. Among the six geographically divided regions, tobacco consumption was reported to be high among the “seven sisters of north-east” (1,2) (9). Differences in cultural practices, means of family support, liking for behaviourally abusive substances, food and drinking habits, etc., distinguish them from citizens of other regions (48). Such behavioral and geographical variations also differentiate most scheduled tribes from other ethnic groups. Parallel to societal differences, a significant proportion of tobacco consumers are found in the lower economic classes, and this has been evident among both genders. Tobacco cessation approaches are meant to create an umbrella for all, but due to area- and tradition-specific diversities, one policy is falling short of dealing with the whole nation. Hence, we feel that now is the time when we should not just think but rather implement region-specific as well as gender-specific strategies that will not disrespect societal standards.

A woman needs to be ready not only physically but also mentally before being bound by the custom of marriage. Her ability to nurture a child develops with her nutritional status on the one hand; on the other hand, knowledge of healthy physical and behavioral practices helps her to accept proper sanitation and WASH habits, an emotional acceptance of the marital relationship, such as a gratifying attitude from her in-laws, willingness for sexual relations with her partner, etc. Women face societal restrictions from accepting formal education after menarche (49) or their childhood is hampered by early marriage, causing psychological distress by making them suffer through the weight of managing a new family, the pressure of being judged by relatives, the hassle of bearing a child right after the marriage, etc., while they should be at school (7). This might cause stress and depression that leave her with no other way but to take refuge in tobacco or any other addictive products (8). Early pregnancy was evident with higher odds of tobacco consumption of both smoking and smokeless types in our study, which was in accordance with other studies as well (50). Earlier, the legal age of marriage for women was 18 years in India. In December 2021, the Union Cabinet agreed to increase this to 21 years. However, the issue of early marriage is prominent enough to date, requiring stricter administrative restrictions to save younger women from falling prey to marriage in the early hours and from untimely pregnancy. Limited awareness about health risks, cultural acceptance of smokeless tobacco, and the use of tobacco as a coping mechanism for stress during early motherhood contribute to this pattern (51).

Mass media, in the form of newspapers, television, and radio, are the most conventional in the Indian scenario. Currently, one of them has reached every household or at least every remote village. The recent addendum to the list is mobile phones, although it is not as thorough as the other options but sufficient to disseminate information or generate awareness. The campaign against tobacco is very common through any of these media, and its effect to reject tobacco during pregnancy was apparent from our analysis. However, the awareness movement could not control for the likelihood of SLT use among pregnant and breastfeeding mothers. A possible reason could be that the majority of the awareness campaigns are directed towards cessation of ST products, while 70% of the Indian students reported being exposed to SLT advertisements (52). Hand-cut khaini, gudaku, and gutkha are quite common in the smallest *paan* shops or tea stalls in India. To protect citizens, stricter age- and gender-specific directives are needed, stating that children, young adults, and women of any age cannot sell any kind of tobacco products. Restricting or banning the production of tobacco dentifrices, called gudaku, which is very common among Indian women (53), could be another step towards de-addiction.

We strongly advocate gender-specific initiatives and effective tobacco cessation counseling by primary healthcare workers for all eligible couples. Strengthening the role of frontline health workers, especially ASHAs, in delivering tailored interventions during the antenatal and postnatal periods could significantly reduce tobacco use among women.

### Strengths and limitations

Tobacco is an addictive substance, and the majority of consumers are aware of its long-term toxic side effects in the long run. Hence, whenever we ask someone if they consume tobacco, there will be many who would hesitate to speak the truth. Therefore, cross-sectional studies lack an exact estimation of sensitive outcomes of interest. Although we include a mammoth proportion of observations from different parts of the nation, the overall estimation becomes close to the actual burden. A nationally representative dataset with a humongous sample size provides these advantages over localized studies. This enhances the external validity and generalizability of the findings to the broader population of pregnant and breastfeeding women in the country. The internal validity is also reasonably robust, as supported by standardized data collection methods. A few factors that we could not include in this study were purchase ability or a proxy indicator for the same as mothers’ occupation due to a considerable number of missing values in the dataset, unavailability of information related to counseling for tobacco cessation during pregnancy, the influence of tobacco at home, and information on second-hand smoking, which might help to understand how a woman gets trapped by tobacco addiction.

Future research should explore why highly educated women continue to use smokeless tobacco, with qualitative studies recommended to understand the underlying motivations, social influences, and barriers to cessation.

## Conclusion

The tobacco menace, especially smokeless tobacco products, is prominently rooted among Indian women of all ages. Addiction at a younger age is not only injurious to the mother but also impedes the development of a child during its intrauterine and postnatal stages. Poor maternal and child health indicates a deficit in healthcare delivery, which is not advantageous for a high-aiming country such as India. Regulation of tobacco production and illicit trade requires substantial reformation and to be dealt with immense thoughtfulness and a strong hand for a fruitful outcome all over the nation.

## Data Availability

Publicly available datasets were analyzed in this study. This data can be found at: https://dhsprogram.com/methodology/survey/survey-display-541.cfm.

## References

[1] Gately I. Tobacco—a cultural history of how an exotic plant seduced civilization. 416 p. Available online at: https://www.google.co.in/books/edition/Tobacco/x41jVocj05EC?hl=en&gbpv=1&dq=1.%09Gately+I.+Tobacco:+a+cultural+history+of+how+an+exotic+plant+seduced+civilization.+Open+Road%2B+Grove/Atlantic%3B+2007+Dec+1.&pg=PA1&printsec=frontcover 12466768

[2] Doll R. Uncovering the effects of smoking: historical perspective. Stat Methods Med Res. (1998) 7:87–117. doi: 10.1177/096228029800700202 9654637

[3] West R McEwen A Bolling K Owen L. Smoking cessation and smoking patterns in the general population: a 1-year follow-up. Addiction. (2001) 96:891–902. doi: 10.1046/j.1360-0443.2001.96689110.x 11399220

[4] Critchley JA Unal B. Health effects associated with smokeless tobacco: a systematic review. Thorax. (2003) 58:435–43. doi: 10.1136/thorax.58.5.435 12728167 PMC1746661

[5] Kaur J Thamarangsi T Rinkoo AV. Regulating smokeless tobacco and processed areca nut in South-East Asia region: the journey so far and the road ahead. Indian J Public Health. (2017) 61:S3–6. doi: 10.4103/ijph.IJPH_242_17, PMID: 28928311

[6] Singh PK Singh L Wehrmeister FC Singh N Kumar C Singh A. Prevalence of smoking and smokeless tobacco use during breastfeeding: a cross-sectional secondary data analysis based on 0.32 million sample women in 78 low-income and middle-income countries. eClin Med. (2022) 53:101660. doi: 10.1016/j.eclinm.2022.101660 PMC9489519 36159043

[7] Islam FMA Walton A. Tobacco smoking and use of smokeless tobacco and their association with psychological distress and other factors in a Rural District in Bangladesh: a cross-sectional study. J Environ Public Health. (2019) 2019:1–11. doi: 10.1155/2019/1424592, PMID: 31885635 PMC6918939

[8] Singh S Jain P Singh PK Reddy KS Bhargava B. White paper on smokeless tobacco & women’s health in India. Indian J Med Res. (2020) 151:513–21. doi: 10.4103/ijmr.IJMR_537_20, PMID: 32719223 PMC7602932

[9] Ghosal S Sinha A Kanungo S Pati S. Declining trends in smokeless tobacco use among Indian women: findings from global adult tobacco survey I and II. BMC Public Health. (2021) 21:2047. doi: 10.1186/s12889-021-12089-6 34753440 PMC8576912

[10] General O of the S. U.S. Department of Health and Human Services. (2006). How tobacco smoke causes disease: The biology and behavioral basis for smoking- attributable disease: A report of the surgeon general. Atlanta, Ga: U.S. department of health and human services, centers for disease control and prevention, national center for chronic disease prevention and health promotion, office on smoking and health, 2010. Available online at: https://www.hhs.gov/surgeongeneral/reports-and-publications/index.html 21452462

[11] Inamdar AS Croucher RE Chokhandre MK Mashyakhy MH Marinho VCC. Maternal smokeless tobacco use in pregnancy and adverse health outcomes in newborns: a systematic review. Nicotine Tob Res. (2015) 17:1058–66. doi: 10.1093/ntr/ntu255, PMID: 25534929

[12] Baba S Wikström AK Stephansson O Cnattingius S. Changes in snuff and smoking habits in Swedish pregnant women and risk for small for gestational age births. BJOG Int J Obstet Gynaecol. (2013) 120:456–62. doi: 10.1111/1471-0528.12067, PMID: 23190416

[13] Napierala M Mazela J Merritt TA Florek E. Tobacco smoking and breastfeeding: effect on the lactation process, breast milk composition and infant development. A critical review. Environ Res. (2016) 151:321–38. doi: 10.1016/j.envres.2016.08.002, PMID: 27522570

[14] Shukla R Kanaan M Siddiqi K. Tobacco use among 1 310 716 women of reproductive age (15–49 years) in 42 low- and middle-income countries: secondary data analysis from the 2010-2016 demographic and health surveys. Nicotine Tob Res. (2021) 23:2019–27. doi: 10.1093/ntr/ntab131, PMID: 34291296 PMC8849114

[15] Lauria L Lamberti A Grandolfo M. Smoking behaviour before, during, and after pregnancy: the effect of breastfeeding. ScientificWorldJournal. (2012) 2012:154910:1–9. doi: 10.1100/2012/154910, PMID: 22536121 PMC3317547

[16] Karmaus W Dobai AL Ogbuanu I Arshard SH Matthews S Ewart S. Long-term effects of breastfeeding, maternal smoking during pregnancy, and recurrent lower respiratory tract infections on asthma in children. J Asthma. (2008) 45:688–95. doi: 10.1080/02770900802178306, PMID: 18951262 PMC2700345

[17] Fried PA. Prenatal exposure to tobacco and marijuana: effects during pregnancy, infancy, and early childhood. Clin Obstet Gynecol. (1993) 36:319–37. doi: 10.1097/00003081-199306000-00011, PMID: 8513627

[18] Deshmukh JS Motghare DD Zodpey SP Wadhva SK. Low birth weight and associated maternal factors in an urban area. Indian Pediatr. (1998) 35:33–6. 9707902

[19] Monawar Hosain GM Chatterjee N Begum A Saha SC. Factors associated with low birthweight in rural Bangladesh. J Trop Pediatr. (2006) 52:87–91. doi: 10.1093/tropej/fmi066 16014761

[20] Pratinidhi A Gandham S Shrotri A Patil A Pardeshi S. Use of ‘Mishri’ a smokeless form of tobacco during pregnancy and its perinatal outcome. Indian J Community Med. (2010) 35:14–8. doi: 10.4103/0970-0218.62547, PMID: 20606913 PMC2888344

[21] Baba S Wikström AK Stephansson O Cnattingius S. Influence of snuff and smoking habits in early pregnancy on risks for stillbirth and early neonatal mortality. Nicotine Tob Res. (2014) 16:78–83. doi: 10.1093/ntr/ntt117, PMID: 23943841

[22] Gupta PC Subramoney S. Smokeless tobacco use and risk of stillbirth: a cohort study in Mumbai, India. Epidemiology. (2006) 17:47. doi: 10.1097/01.ede.0000190545.19168.c4 16357594

[23] Can Özalp E Yalçın SS. Is maternal cigarette or water pipe use associated with stopping breastfeeding? Evidence from the Jordan population and family health surveys 2012 and 2017–18. Int Breastfeed J. (2021) 16:43. doi: 10.1186/s13006-021-00387-z 34053454 PMC8165988

[24] Logan CA Rothenbacher D Genuneit J. Postpartum smoking relapse and breast feeding: defining the window of opportunity for intervention. Nicotine Tob Res. (2017) 19:367–72. doi: 10.1093/ntr/ntw224 27613913

[25] Bahadori B Riediger ND Farrell SM Uitz E Moghadasian MF. Hypothesis: smoking decreases breast feeding duration by suppressing prolactin secretion. Med Hypotheses. (2013) 81:582–6. doi: 10.1016/j.mehy.2013.07.007, PMID: 23948597

[26] Nordhagen LS Kreyberg I Bains KES Carlsen KH Glavin K Skjerven HO. Maternal use of nicotine products and breastfeeding 3 months postpartum. Acta Paediatr. (2020) 109:2594–603. doi: 10.1111/apa.15299, PMID: 32274823

[27] Macchi M Bambini L Franceschini S Alexa ID Agostoni C. The effect of tobacco smoking during pregnancy and breastfeeding on human milk composition—a systematic review. Eur J Clin Nutr. (2021) 75:736–47. doi: 10.1038/s41430-020-00784-3, PMID: 33087893

[28] Burianova I Bronsky J Pavlikova M Janota J Maly J. Maternal body mass index, parity and smoking are associated with human milk macronutrient content after preterm delivery. Early Hum Dev. (2019) 137:104832. doi: 10.1016/j.earlhumdev.2019.104832, PMID: 31422343

[29] Mennella JA Yourshaw LM Morgan LK. Breastfeeding and smoking: short-term effects on infant feeding and sleep. Pediatrics. (2007) 120:497–502. doi: 10.1542/peds.2007-0488, PMID: 17766521 PMC2277470

[30] Mahar I Bagot RC Davoli MA Miksys S Tyndale RF Walker CD. Developmental hippocampal neuroplasticity in a model of nicotine replacement therapy during pregnancy and breastfeeding. PLoS One. (2012) 7:e37219. doi: 10.1371/journal.pone.0037219, PMID: 22615944 PMC3352874

[31] Nakauchi S Malvaez M Su H Kleeman E Dang R Wood MA. Early postnatal nicotine exposure causes hippocampus-dependent memory impairments in adolescent mice: association with altered nicotinic cholinergic modulation of LTP, but not impaired LTP. Neurobiol Learn Mem. (2015) 118:178–88. doi: 10.1016/j.nlm.2014.12.007, PMID: 25545599 PMC4331249

[32] Narayanan U Birru S Vaglenova J Breese CR. Nicotinic receptor expression following nicotine exposure via maternal milk. Neuroreport. (2002) 13:961–3. doi: 10.1097/00001756-200205240-00012, PMID: 12004199

[33] Letourneau AR Sonja B Mazure CM O’Malley SS James D Colson ER. Timing and predictors of postpartum return to smoking in a Group of Inner-City Women: an exploratory pilot study. Birth. (2007) 34:245–52. doi: 10.1111/j.1523-536X.2007.00177.x, PMID: 17718875

[34] Singh PK Jain P Singh N Singh L Singh S. Smokeless tobacco use among pregnant women in India: the tale of two nationally representative surveys. Asian Pac J Cancer Prev. (2022) 23:389–92. doi: 10.31557/APJCP.2022.23.2.389, PMID: 35225448 PMC9272601

[35] Thakore D Chavda M Parmar G Sheth T Shah M. Tobacco use and women in India. Int J Sci Stud. (2021) 9:11–5.

[36] Mohan P Lando HA Panneer S. Assessment of tobacco consumption and control in India. Indian J Clin Med. (2018) 9:1179916118759289. doi: 10.1177/1179916118759289, PMID: 40372777

[37] Mallick J Satpathy S. Estimation of women beedi workers in India and their socio- economic condition. Ind J Labour Econ. (2021) 64:499–521. doi: 10.1007/s41027-021-00320-2

[38] Hung HJ Chen CY Wang SL Wu TN Lee CH Cheng SY. Environmental tobacco smoke: relationship to early pregnancy discomforts. Am J Health Behav. (2017) 41:320–8. doi: 10.5993/AJHB.41.3.11, PMID: 28376976

[39] Nair S Schensul JJ Begum S Pednekar MS Oncken C Bilgi SM. Use of smokeless tobacco by Indian women aged 18–40 years during pregnancy and reproductive years. PLoS One. (2015) 10:e0119814. doi: 10.1371/journal.pone.0119814, PMID: 25786247 PMC4364978

[40] Krishnamoorthy Y Ganesh K. Spatial pattern and determinants of tobacco use among females in India: evidence from a nationally representative survey. Nicotine Tob Res. (2020) 22:2231–7. doi: 10.1093/ntr/ntaa137, PMID: 32722803

[41] Solhi M Fattahi E Manzari ZS Gupta PC Kargar M Kasmaei P. The reasons for using smokeless tobacco: a review. Iran J Public Health. (2021) 50:492–501. doi: 10.18502/ijph.v50i3.5589 34178796 PMC8214603

[42] Bottorff JL Haines-Saah R Kelly MT Oliffe JL Torchalla I Poole N. Gender, smoking and tobacco reduction and cessation: a scoping review. Int J Equity Health. (2014) 13:1–5. doi: 10.1186/s12939-014-0114-2 25495141 PMC4297403

[43] Iqbal N Awan S Riaz U Khan MW Bana S Fazal M. Difference in gender perception regarding smokeless tobacco consumption; a cross sectional survey from Karachi Pakistan. Eur Respir J (2019);54:PA706. Available online at: https://publications.ersnet.org/content/erj/54/suppl_63/PA706

[44] Singh S Jain R Joshi I Chandra R Singh L Singh PK. Determinants of initiation, continuation and cessation of smokeless tobacco use among pregnant and lactating women: a qualitative study from low-income communities in urban India. Health Policy Plan. (2023) 38:907–15. doi: 10.1093/heapol/czad056, PMID: 37494416

[45] Subramanian SV Nandy S Kelly M Gordon D Smith GD. Patterns and distribution of tobacco consumption in India: cross sectional multilevel evidence from the 1998-9 national family health survey. BMJ. (2004) 328:801–6. doi: 10.1136/bmj.328.7443.801, PMID: 15070637 PMC383376

[46] Singh JK Acharya D Kadel R Adhikari S Lombard D Koirala S. Factors associated with smokeless tobacco use among pregnant women in rural areas of the southern Terai, Nepal. J Nepal Health Res Counc (2017);15:12–19. Available online at: https://pubmed.ncbi.nlm.nih.gov/28714486/28714486 10.3126/jnhrc.v15i1.18007

[47] Persai D Panda R Mathur MR. Self-reported practices and attitudes of community health workers (accredited social health activist) in tobacco control – findings from two states in India. Int J Prev Med. (2015) 6:48. doi: 10.4103/2008-7802.158177 26124945 PMC4462774

[48] Yadav J Singh JK Gautam S. Correlates of substance use in northeast states, India. Int J Community Med Public Health. (2016) 3:1531–9. doi: 10.18203/2394-6040.ijcmph20161623

[49] Seth R Bose V Qaiyum Y Chandrashekhar R Kansal S Taneja I. Social determinants of child marriage in rural India. Ochsner J. (2018) 18:390–4. doi: 10.31486/toj.18.0104, PMID: 30559625 PMC6292470

[50] Datta BK Tiwari A Fazlul I. Child marriage and risky health behaviors: an analysis of tobacco use among early adult and early middle-aged women in India. BMC Womens Health. (2022) 22:206. doi: 10.1186/s12905-022-01781-3 35655201 PMC9164419

[51] Sharma R.. Smokeless tobacco use among women of reproductive age and during pregnancy in low and middle income countries-distribution and sociocultural characteristics (doctoral dissertation, University of York)

[52] Mehrotra R Yadav A Sinha DN Parascandola M John RM Ayo-Yusuf O. Smokeless tobacco control in 180 countries across the globe: call to action for full implementation of WHO FCTC measures. Lancet Oncol. (2019) 20:e208–17. doi: 10.1016/S1470-2045(19)30084-1, PMID: 30942182

[53] Sinha DN Palipudi KM Gupta PC Singhal S Ramasundarahettige C Jha P. Smokeless tobacco use: a meta-analysis of risk and attributable mortality estimates for India. Indian J Cancer. (2014) 51:S73–7. doi: 10.4103/0019-509X.147477 25526253

[54] Adekola AD Alli OI Mbata AO Ogbeta CP. Integrating multisectoral strategies for tobacco control: evidence-based approaches and public health outcomes.

[55] Bertani AL Garcia T Tanni SE Godoy I. Preventing smoking during pregnancy: the importance of maternal knowledge of the health hazards and of the treatment options available. J Bras Pneumol. (2015) 41:175–81. doi: 10.1590/S1806-37132015000004482, PMID: 25972970 PMC4428855

