# Supplementary material for: Prevalence and factors associated with tobacco use among pregnant and breastfeeding mothers in India: insights from the National Family Health Survey-5 (2019–21)
Source: Front Public Health. 2025 Jun 6;13:1495522. doi: 10.3389/fpubh.2025.1495522 (PMC12180415; doi:10.3389/fpubh.2025.1495522)
Supplement: Supplementary file 1 [file Table_1.docx]

**Supplementary Table-S1:Comparison for socio-demographic characteristics between currently pregnant or lactating and mothers who are not pregnant or not lactating at the time of interview.**

| **Socio demographic characteristics** | | **Currently Pregnant and/or Lactating mothers n(%)** | **Currently non-pregnant and non- lactating mothers n(%)** |
| --- | --- | --- | --- |
| **Age** | Mean (± SD) | 26.33 (±4.88) | 31.28 (±10.45) |
|  | Median (IQR) | 26 (23-29) | 32 (21-40) |
| **Mothers age at first delivery** | Lower age group  (13-20 years) | 59345 (46.91) | 350509 (59.21) |
|  | Higher age group  (21-45 years) | 67152 (53.09) | 241434 (40.79) |
| **Residence** | Urban | 25133 (19.30) | 154253 (26.03) |
|  | Rural | 105101 (80.70) | 438446 (73.97) |
| **Education** | No formal education | 25517(19.59) | 142288 (24.01) |
|  | Primary | 15503 (11.90) | 68351 (11.53) |
|  | Junior High | 23389 (17.96) | 94790 (15.99) |
|  | Secondary | 27652 (21.23) | 124356 (20.98) |
|  | Higher Secondary | 18967 (14.56) | 80335 (13.55) |
|  | Above higher sec | 19206 (14.75) | 82579 (13.93) |
| **Ethnicity** | SC | 26701 (21.78) | 113034 (20.12) |
|  | ST | 26744 (21.82) | 108097 (19.24) |
|  | OBC | 48843 (39.85) | 227737 (40.54) |
|  | Others | 20282 (16.55) | 112928 (20.10) |
| **Occupation** | Currently working | 3326 (16.98) | 24977 (28.06) |
|  | Currently not working | 16267 (83.02) | 64025 (71.94) |
| **Wealth Index** | Poorest | 35138 (26.98) | 114161 (19.26) |
|  | Poorer | 31033 (23.83) | 128986 (21.76) |
|  | Middle | 25483 (19.57) | 125849 (21.23) |
|  | Richer | 21720 (16.68) | 117791 (19.87) |
|  | Richest | 16860 (12.95) | 105912 (17.87) |
| **Region** | North | 15846 (12.17) | 88683 (14.96) |
|  | Central | 41504 (31.87) | 171247 (28.89) |
|  | East | 27214 (20.90) | 90814 (15.32) |
|  | North east | 20828 (15.99) | 82250 (13.88) |
|  | West | 10289 (7.90) | 61462 (10.37) |
|  | South | 14553 (11.17) | 98243 (16.58) |
| **Alcohol consumption** | Almost everyday | 232 (0.18) | 1558 (0.26) |
|  | Once a week | 755 (0.58) | 4533 (0.76) |
|  | Less than once a week | 1056 (0.81) | 5355 (0.90) |
|  | Never consumes | 128191 (98.43) | 581253 (98.07) |
| **Exposure to Mass-media** | To some extent | 93121 (71.50) | 458316 (77.33) |
|  | Not at all | 37113 (28.50) | 134383 (22.67) |
